# Correlation between the Expression of Topo IIα and Ki67 in breast cancer and its clinical Pathological characteristics

**DOI:** 10.12669/pjms.334.13143

**Published:** 2017

**Authors:** Jihai Jin, Dongxing Zheng, Yujuan Liu

**Affiliations:** 1 Jihai Jin Department of Breast Surgery, Binzhou People’s Hospital, Shandong 256600, China; 2 Dongxing Zheng, Department of Oncology II, Binzhou People’s Hospital, Shandong 256600, China; 3 Yujuan Liu, Department of Pediatrics, Binzhou People’s Hospital, Shandong 256600, China

**Keywords:** Breast cancer, Pathological characteristics, Topo IIα

## Abstract

**Objective:**

To investigate the expression of Topo IIα and Ki67 and its clinical significance.

**Methods:**

The clinical pathological data of one hundred and sixteen invasive breast cancer patients who were admitted into our hospital from July 2013 to December 2015 and underwent radical mastectomy were retrospectively analyzed. The expression of topoisomerase (Topo) IIα and Ki67 was detected using immunohistochemical method, and the correlation between the two kinds of proteins and the general clinical pathological characteristics of the patients was analyzed.

**Results:**

The positive expression rates of Topo IIα and Ki67 in breast cancer were 58.6% and 75% respectively. The expression of Topo IIα was in no apparent correlation with the age, tumor size, estrogen receptor (ER), progesterone receptor (PR) and human epidermal growth factor receptor 2 (HER-2) (P>0.05), but in a correlation with the number of metastatic lymph glands (P<0.05). The expression of Ki67 was in no apparent correlation with the age, tumor size, EP and HER-2, but in a correlation with the number of metastatic lymph glands and PR (P<0.05). The multi-factor logistic regression analysis results suggested that the number of metastatic lymph glands was the independent predictive factor of Topo IIα positive expression and the number of metastatic lymph glands and PR protein expression state are the independent predictive factors of Ki67 positive expression.

**Conclusion:**

Topo IIα and Ki67 can be regarded as the indicators for reflecting the proliferation activity of tumor cells, and the detection of Topo IIα and Ki67 expression is of great significance to the prognosis evaluation of breast cancer patients and clinical treatment.

## INTRODUCTION

Breast cancer as a common malignant tumor has a high incidence among females, which can severely affect the health of human. The therapies of breast cancer include surgery, chemotherapy and radiotherapy, but few breast cancer patients survive after surgery, which is attributed to the occurrence of breast cancer metastasis or late clinical stage.^1^-^3^ Therefore, study on the molecular biological characteristics of breast cancer patients is of great significance to the determination of prognosis and the formulation of clinical chemotherapy regimens.

Recent studies have found that, multiple biological markers are in a close correlation with the prognosis of breast cancer.^4^,^5^ Topoisomerase (Topo) IIα and Ki67 have been proved involving in the formation and development of tumor and can be regarded as the independent indicators for guiding clinical quality and indicating prognosis. Topo IIα which is TOP II gene encoded DNA topoisomerase is an important ribozyme for DNA replication and transcription. Topo IIα is the target enzyme of anthracyclines, and its overexpression can reflect the proliferation state of cells.^6^ Ki67 as the proliferating cell nuclear antigen (PCNA) for determining the proliferation state of tumor cells and prognosis has been extensively applied in clinics.^7^

This study aims to investigate the correlation between the expression of Ki67 and Topo IIα and the clinical pathological indicators of patients with breast cancer through detecting the expression of Ki67 and Topo IIα in the breast cancer tissues of 116 breast cancer patients who were admitted into our hospital, with the intention of providing a basis for the clinical treatment and prognosis determination.

## METHODS

One hundred and sixteen patients who underwent radical mastectomy in our hospital from July 2013 to December 2015 were selected as the research subjects. Those who had complete pathological results, immunohistochemical results and clinical data after surgery, underwent no chemotherapy, radiotherapy and targeted treatment before surgery, and had no cardiovascular and cerebrovascular diseases; infection and hematological system diseases were included. This study was reviewed and approved by the ethics committee of our hospital. All the included patients signed informed consent.

### Experimental results

All the specimens were fixed by 10% neutral buffered formalin, embedded by paraffin, and sliced into sections in a thickness of 4 μm. Then the expression of Topo IIα and Ki67 was detected using immunohistochemical En Vision method. Phosphate buffer solution was taken as negative controls instead of first antibody. The positive sections were taken as positive controls. They were observed under a light microscope. The experimenters operated rigorously according to the instructions on the kit. All the reagents were purchased from Wuhan Boster Biological Technology Co., Ltd., Hubei, China.

### Results determination

If the tumor cell nucleus was dark brown and percentage of positive cells was larger than 10%, Ki-67 was determined as positive; otherwise, it was determined as negative. If the cytoplasm of the positive cells was in dark brown, Topo IIα was determined as positive. The percentage of positive cells lower than 30% was scored for one point, between 30% and 75% as two points, and higher than 75% as three points. No staining of cytomembrane was scored for 0 point, incomplete staining of cytomembrane and percentage of positive cells less than 10% was scored for one point, complete staining of cytomembrane and percentage of positive cells less than 10% for two point, and complete staining of cytomembrane or percentage of positive cells no less than 10% was determined as three points. If the product of percentage of positive cells and positive strength was no less than three points, the result was determined as positive. The results were determined by two pathologists who have been entitled as associate chief physician or higher. The third pathologist was needed if the results were inconsistent.

### Statistical processing

Data were analyzed using SPSS ver. 21.0. Enumeration data were compared using Chi-square test. Fisher’s exact test was used for statistical analysis if sample theoretical frequency was less than one. The indicators with statistical significance were processed by multi-factor logistic regression analysis additionally to screen independent predictive indicators. Difference was considered as statistically significant if P<0.05.

## RESULTS

### Expression of Topo IIα and Ki67 in breast cancer

**Fig. 1:**
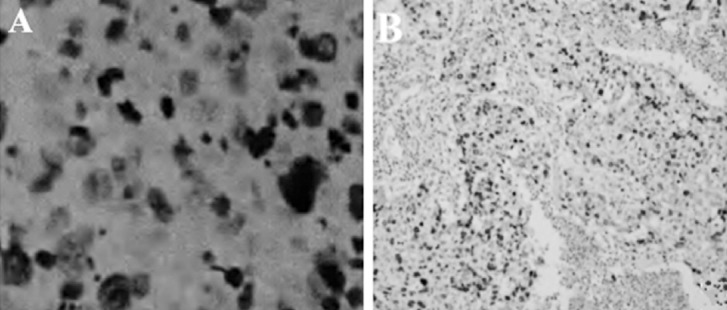
The expression of Topo IIα and Ki67 (immunohistochemical, ×100): A: positive expression of Ki67; B: positive expression of Topo Iiα CI: confidence interval

The positive expression rates of Topo IIα and Ki67 in the breast cancer tissues from 116 cases were 58.6% (68/116) and 76% (88/116) respectively. The positive expression of Topo IIα and Ki67 manifested as brown yellow substances in cytoplasm under the microscope, as shown in Fig. 1.

### The correlation between Topo IIα and Ki67 expression and clinical pathological characteristics

The positive expression of Topo IIα was in a correlation with the number of metastatic lymph glands, and the difference had statistical significance (X^2^=7.917, P=0.043); but it was in no correlation with age, tumor size, ER, PR and HER-2 (P>0.05; Table-I). The positive expression of Ki67 was in a correlation with the number of metastatic lymph glands and PR protein expression, and there were statistically significant differences (X^2^=8.302, P=0.032; X^2^=11.965, P=0.000; Table-I).

**Table-I:** The correlation between Topo IIα and Ki67 expression and clinical pathological characteristics.

*Factor*		*N*	*Topo IIα*			*Ki67*		

			*+*	*-*	*P*	*+*	*-*	*P*
Age (years)	<50	53	25	28	0.837	38	15	0.326
	≥50	63	30	33		39	24	
Tumor size (cm)	≤2	30	11	19	0.097	18	12	0.076
	>2, ≤5	71	41	30		53	18	
	>5	15	5	10		6	9	
Number of metastatic lymph glands	0	59	21	38	0.043	35	24	0.037
	≥1, <4	18	10	8		9	9	
	≥4, <10	15	9	8		15	0	
	≥10	24	18	6		19	5	
PR	+	56	27	29	0.829	18	38	0.000
	-	60	31	29		27	33	
ER	+	74	36	38	0.868	46	28	0.238
	-	42	21	21		31	11	
HER-2	+	45	25	20	0.269	33	12	0.242
	-	71	31	40		44	27	

### Multi-factor logistic regression analysis on Topo IIα and Ki67

Number of metastatic lymph glands and PR protein expression which suggested statistically significant differences in the single-factor analysis was processed by multi-factor logistic regression analysis. The results indicated that number of metastatic lymph glands was the independent predictive factor for the positive expression of Topo IIα, and number of metastatic lymph glands and PR protein expression were the independent predictive factors for the positive expression of Ki67 (Table-II).

**Table-II:** Multi-factor logistic regression analysis on Topo IIα and Ki67 expression in breast cancer tissues.

*Factor*	*Topo IIα*			*Ki67*		

	*P*	*Odd ratio*	*95% CI*	*P*	*Odd ratio*	*95% CI*
Number of metastatic lymph glands	0.006	0.872	1.175-2.700	0.028	1.734	1.051-2.856
PR	0.751	0.858	0.398-1.927	0.000	0.127	0.037-0.404

CI: confidence interval.

## DISCUSSION

Breast cancer is a multi-factor and multi-molecular disease with protein abnormality. Multiple molecular markers such as Topo, ER, PR, Cerb B-2 and Ki67 are all involved in the occurrence and development of breast cancer, and their positive expression is of great significance to clinical medication and prognosis prediction.^8^ TOPO existing in cell nucleus can change the topological forms of DNA (I and II) by catalyzing DNA breakage and binding of DNA chain.^9^ Topo II, also called gyrase, includes two isozymes, i.e., α and β. Topo IIα is not only a key enzyme in DNA replication process but also a targeted enzyme which anthracyclines act on. Topo IIα with cell cycle specificity increases sharply at the cell cycle stage S-G2/M and decreases after mitosis (stage G1 and G0). Positive expression of Topo II indicates tumor cells are in a proliferative state; hence it can be used for reflecting the proliferation activity of tumor cells.

This study suggested that the positive rate of Topo IIα was 58.6%, which was in no apparent correlation with age, tumor size, ER, PR and HER-2 and in a correlation with number of metastatic lymph glands; the positive expression rate of Topo IIα became higher with the increase of the number of metastatic lymph glands.

Single-factor and multi-factor logistic regression analysis results both indicated that number of metastatic lymph glands was the independent predictive indicator for the positive expression of Topo IIα. Number of metastatic glands is usually a key factor used for evaluating the prognosis of invasive breast cancer; the larger the number of metastatic lymph glands, the later the breast cancer stage and the poorer the prognosis.^10^ Therefore, it was assumed that Topo IIα as the important indicator for reflecting the proliferation activity of tumor cells was in a correlation with the malignancy progress and metastasis of breast cancer as well as prognosis.

Ki67, the proliferating cell nuclear antigen, exists in all the stages except G0. Moreover, it is crucial in the biological and pathological death of cells. Therefore, tumors will be induced or the occurrence of tumors will be accelerated once the effect of Ki67 is inhibited.^11^-^13^ Studies have pointed out that Ki67 was correlated to many tumors such as breast cancer,^14^,^15^ non-small cell lung cancer, non-Hodgkin’s lymphoma, laryngeal squamous cancer, epithelial ovarian cancer, bladder cancer, cervical cancer, anaplastic oligodendroglioma and colon cancer. A previous study reported that the expression of Ki67 was in no obvious correlation with the age and tumor size of patients.^16^ However, the correlation between Ki67 expression and lymph glands metastasis is controversial. In this experiment, the positive rate of Ki67 was 76%, which was in no apparent correlation with age, tumor size, ER and HER-2, but in a correlation with the number of lymph nodes and PR. The multi-factor logistic regression analysis results indicated that number of metastatic lymph glands and PR were the independent predictive indicators for the positive expression of Ki67 and that Ki67 was in a close correlation with the occurrence and development of breast cancer and prognosis. Zhang HY et al. found that the coexpression of Topo IIα and Ki-67 could be taken as the prediction indicator for the efficacy of chemotherapy using anthracyclines in the treatment of HER-2-positive breast carcinoma, i.e., breast cancer patients with positive Topo IIα and Ki-67 were more sensitive to chemotherapy and benefited more after chemotherapy.^17^

## CONCLUSION

In conclusion, Topo IIα and Ki67 are the immunohistochemical indicators for evaluating the treatment efficacy and prognosis of breast cancer. The positive expression of Ki67 in breast cancer tissues can be regarded as the prognosis determination indicator of breast cancer. The expression of Topo IIα can be regarded as an important reference indicator for guiding chemotherapy for breast cancer. The joint detection of Topo IIα and Ki67 is beneficial to the prognosis determination of breast cancer and the selection of postoperative therapeutic regimen, and the coexpression of Topo IIα and Ki67 can be used for predicting chemotherapeutic efficacy.

### Authors’ Contribution

**JHJ:** Study design, data collection and analysis.

**DXZ &YJL:** Manuscript preparation, drafting and revising.

**JHJ &YJL:** Review and final approval of manuscript.
